# Advancement in knowledge and skills of nursing students in operation theatre procedures with mobile based learning

**DOI:** 10.17533/udea.iee.v42n2e15

**Published:** 2024-07-09

**Authors:** Ahrar Ahmed Dev, Kanika Rai, Amoldeep Sharma, Jyoti Sarin

**Affiliations:** 1 RN, MSN. Nursing Tutor. Email: ahrardev11@gmail.com Govt Nursing College, Doda India ahrardev11@gmail.com; 2 RN, Ph.D. Professor-cum-Vice Principal. Email: nehukanu@gmail.com, kanika@chitkara.edu.in. Corresponding author Chitkara University India nehukanu@gmail.com; 3 RN, Community Health Nursing. Associate Professor. Email: amolsharma206@gmail.com Maharishi Markandeshwar University Community Health Nursing India amolsharma206@gmail.com; 4 RN, Ph.D. Principal. Email: sarinjyoti@yahoo.co.in Maharishi Markandeshwar University India sarinjyoti@yahoo.co.in; 5 Govt Nursing College, Doda, Jammu and Kashmir; India Govt Nursing College, Doda Govt Nursing College, Doda Jammu and Kashmir India; 6 Chitkara School of Health Sciences, Chitkara University, Punjab; India Chitkara University Chitkara School of Health Sciences Chitkara University Punjab India; 7 College of Nursing at Maharishi Markandeshwar (deemed to be university), Mullana, Ambala, Haryana; India. Maharishi Markandeshwar University College of Nursing Maharishi Markandeshwar Mullana Ambala India

**Keywords:** learning, knowledge, clinical competence, smartphone, operating rooms, students, nursing, aprendizaje, conocimiento, competencia clínica, teléfono inteligente, quirófanos, estudiantes de enfermería, aprendizagem, conhecimento, competência clínica, smartphone, salas cirúrgicas, estudantes de enfermagem

## Abstract

**Objective.:**

To evaluate the effectiveness of mobile -based learning (MBL) in improving nursing students' knowledge and skills when performing procedures in the operating room.

**Methods.:**

A quasi-experimental study with control group, pre- and post-intervention assessment was conducted. A total of 128 nursing students from India were recruited by purposive sampling and randomly assigned to the intervention (use of a telephone application containing videos on hand washing, surgical gown donning, gloving, and assisting during intubation) and conventional education groups. A validated Structured Knowledge Questionnaire and an Objective Structured Clinical Examination (OSCE) scale was used to assess nursing students' competencies in relation to operating room procedures and a mobile-based learning satisfaction opinion questionnaire was administered.

**Results.:**

The findings showed that the improvement in the mean knowledge and skills score was greater in the intervention group than in the control group (p<0.001). The administration of the MBL was rated as highly satisfactory by 93.8% of the students exposed to this learning method.

**Conclusion.:**

The MBL intervention was effective in improving nursing students' knowledge and skills in the evaluated operating room procedures.

## Resumen

**Objetivo.:**

Evaluar la eficacia del aprendizaje basado en dispositivos móviles (MBL) para mejorar los conocimientos y habilidades de los estudiantes de enfermería a la hora de realizar procedimientos en el quirófano.

**Método.:**

Se realizó un estudio cuasiexperimental con grupo de control y evaluación previa y posterior a la intervención. Un total de 128 estudiantes de enfermería de la India fueron reclutados mediante muestreo intencional y asignados aleatoriamente a los grupos de intervención (uso de una aplicación telefónica que contenía vídeos sobre el lavado de manos, la colocación de la bata quirúrgica, la colocación de guantes y la asistencia durante la intubación) y de educación convencional. Se utilizó un cuestionario validado de conocimientos estructurados y una escala de examen clínico estructurado objetivo (OSCE) para evaluar las competencias de los estudiantes de enfermería en relación con los procedimientos de quirófano, y se administró un cuestionario de opinión sobre la satisfacción del aprendizaje basado en el móvil.

**Resultados.:**

Los resultados mostraron que la mejora en la puntuación media de conocimientos y habilidades fue mayor en el grupo de intervención que en el grupo de control (p<0.001). La administración del MBL fue calificada como altamente satisfactoria por el 93.8% de los estudiantes expuestos a este método de aprendizaje.

**Conclusión.:**

La intervención MBL fue eficaz para mejorar los conocimientos y habilidades de los estudiantes de enfermería en los procedimientos de quirófano evaluados.

## Resumo

**Objetivo.:**

Avaliar a eficácia da aprendizagem baseada em dispositivos móveis (MBL) na melhoria do conhecimento e das habilidades dos estudantes de enfermagem ao realizar procedimentos na sala de cirurgia.

**Métodos.:**

Foi realizado um estudo quase experimental com grupo controle, com avaliação antes e após a intervenção. 128 estudantes de enfermagem de Ambala (Índia) foram recrutados por amostragem proposital e distribuídos aleatoriamente nos grupos de intervenção (uso de um aplicativo telefônico que continha vídeos sobre: ​​lavagem das mãos, como colocar a bata cirúrgica, luvas e assistência durante a intubação) e educação convencional. A escala OSCE (Objective Structured Clinical Examination, em inglês) foi utilizada para avaliar as competências dos estudantes de enfermagem em relação aos procedimentos do centro cirúrgico e foi aplicado um questionário de opinião sobre a satisfação de aprender com base em dispositivos móveis.

**Resultados.:**

Os resultados mostraram que a melhoria na pontuação média de conhecimentos e habilidades foi maior no grupo de intervenção do que no grupo de controle (p<0.001). A aplicação do MBL foi avaliada como altamente satisfatória por 93.8% dos alunos expostos a esse método de aprendizagem.

**Conclusão.:**

A intervenção baseada no MBL foi eficaz na melhoria do conhecimento e das habilidades dos estudantes de enfermagem nos procedimentos avaliados na sala de cirurgia.

## Introduction

The significance of clinical nursing practice is on par with that of theoretical nursing education. In recent years, there has been a development of various nursing practice contents aimed at providing nursing students with indirect exposure to clinical situations.^1^ Mobile-based learning is anticipated to have a significant impact on collaborative learning due to its widespread appeal. The ubiquity of mobile phones has facilitated seamless connectivity among users, hence fostering interactive learning experiences. The utilization of educational technology, such as Mobile Based Learning, is widely acknowledged as a means to enhance theoretical knowledge and foster skill development.^2^ The operation theatre complex is considered the key component of any significant surgical facility, where various procedures and surgeries are performed. In the United Kingdom, operation theatres were named so because they historically featured semi-circular amphitheaters that enabled students to observe medical and nursing procedures.^3^

The specialized operating room nursing officer is commonly acknowledged as the sole healthcare professional possessing the requisite expertise to oversee asepsis, instruments, infections, complications, as well as the control and management of biological specimens in the context of surgical procedures. In order to provide nursing care during surgical procedures in the operating theatre, operation theatre nurses must possess specialized knowledge and skills. The competence of operating theatre nurses is essential to guarantee patient safety during surgical procedures.^4^ The nurses working in the operation theatre complex (as a scrub/ circulating nurse) are required to perform four essential technical competencies, including gowning and gloving, setting up instruments prior to surgery, ensuring that the patient is draped appropriately, and that sterility is well maintained in the sterilized area throughout the procedure.^5^ The surgical and anesthetic team members involved in a perioperative intervention or procedure perform these procedures. Scrubbing, donning a gown, and donning gloves should occur immediately after surgical hand antisepsis. The application of antiseptic hand wash prior to donning sterile garments before a surgical procedure is referred to as surgical hand antisepsis.^6^

According to estimates, a significant number of individuals, ranging from 4.5 to 5.7 billion, experience SSI annually as a result of inadequate aseptic technique measures in hospitals. A study reported that around 9% of hospital patients in India develop healthcare-associated infections, particularly post-operative infections, leading to an estimated 5,000-15,000 deaths annually. The significance of enhancing the understanding and implementation of aseptic technique among theatre nurses was also suggested.^7^ The removal of personal protective equipment (PPE) by healthcare personnel can lead to contamination of their skin and clothing, which increases the likelihood of infection and the dissemination of pathogens. It has been found that educational interventions have the potential to improve the aseptic technique and the donning and doffing of surgical gown, which can ultimately lead to a reduction in contamination.^8^

Mobile phones are anticipated to have a significant impact on collaborative learning due to their widespread use and ability to keep users connected, leading to increased opportunities for interactive learning. Furthermore, mobile phones possess educational potential in facilitating discussions pertaining to teaching and learning methodologies.^9^ Internet prevalence is increasing daily, which increases the potential for online study in a direct or indirect manner.^10^ In the age of technology, the educational system is evolving and transforming towards experiential and comprehensive learning aids.^11^ Mobile-based education has the potential to offer a self-directed learning environment, particularly to the student nurses, enabling them to access information anytime, anywhere and also practice skills repeatedly, without any constraints of space and time.^12^ It also has the potential to offer a non-judgmental learning environment, allowing students to engage in repeated practice sessions without feeling apprehensive about making mistakes. ^9^ It is possible that nursing students experience stress when acquiring and implementing nursing techniques in a laboratory or medical settings.^13^ Additional factors that have been reported to contribute to non-adherence with standard precautions are inadequate comprehension and awareness among healthcare personnel regarding the appropriate usage of protective barriers and insufficient training.^14^ Nowadays, technology-enhanced learning, particularly using mobile devices, has the potential to be a valuable tool in educating younger generations.^15^

Although there are procedures for scrubbing, donning a gown, and donning gloves, there appears to be a great deal of deviation among the operating room staff, which may be the result of individual training/ experience, workload, time constraint or lack of reinforcement for adherence to protocols. To maintain standardized operating procedures across the surgical team, continuous research on this topic is necessary.^16^ Students are enthusiastic about m-learning interventions in a study on the use of portable electronic gadgets for teaching. M-learning and the web give learners an "open-door policy" to learning resources, allowing them to access these resources from any location and at any time zone, which they can utilize repeatedly.^17^ Although there are numerous benefits of utilizing Mobile Based Learning in terms of education, there is a paucity of research on this pedagogical approach, particularly in nursing education, when it comes to teaching skills related to operation theatre. Hence, the objective of this research was to find out how effective is Mobile based learning in augmenting the knowledge and skills of nursing students in performing operation theatre procedures.

## Methods

**Figure 1:**
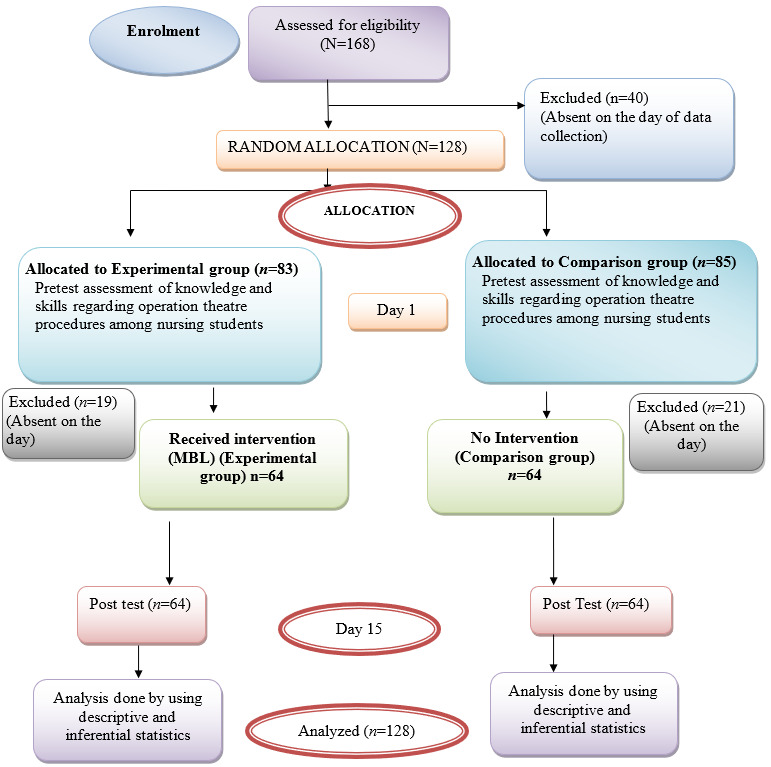
CONSORT diagram for sample selection

The research employed a Quasi-Experimental design with a non-equivalent control group and pre-test, post-test measurements. The study was carried out at M.M College of Nursing and M.M. Super-specialty Hospital located in Mullana, Ambala, Haryana between 2020-2021. A total of 128 study participants (64 in each group) were recruited using purposive sampling. A power analysis was conducted using the Cohen's d formula based on previous research evidences for intervention studies among nursing students. Since the number of students in both the programs i.e., B.Sc. Nursing and Post Basic B.Sc. Nursing differ, quota sampling was used to ascertain the number of students from both programs in order to increase the representativeness of groups. In addition, the random assignment of nursing students was performed using a computer-generated random number method. On day one, after the pre-assessment of knowledge via google form and skills by OSCE, MBL was introduced to the experimental group. OSCE was conducted by peer experts after pre-briefing sessions with them and establishing reliability for evaluating the procedures. The researcher assisted the students in installing the application on their mobile devices and demonstrated how to access the application's content and view videos. Any uncertainties or questions were resolved. The experimental set of students had an access to the application for two weeks. (Figure 1) The students in the conventional group received no intervention but were exposed to regular classes and demonstrations regarding OT procedures. Post-test was taken on 15^th^ day after the intervention in both the groups. Satisfaction regarding MBL was assessed using a semantic differential scale shared via google form.

Inclusion & Exclusion criteria. The study included those nursing students who were willing to participate, having their own Smartphone and available during data collection. The nursing students who were absent on three consecutive days and those who did not receive the allocated intervention were excluded from the study. 

Ethical consideration. To conduct the final investigation, ethical approval was obtained from the Institutional Ethical Committee (IEC-1506). Prior permission from the principals of respective nursing colleges was taken for the final study. Participants were recruited in the study only after receiving written informed consent and assurances that their responses would be kept confidential. 

### Development of Intervention

**Figure 2:**
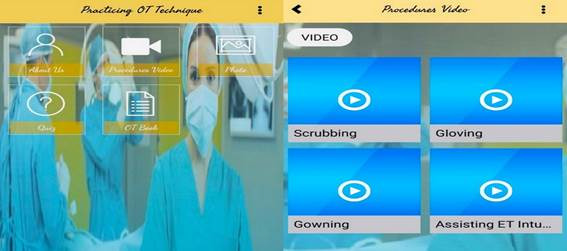
Interface of the mobile application

**Figure 4:**
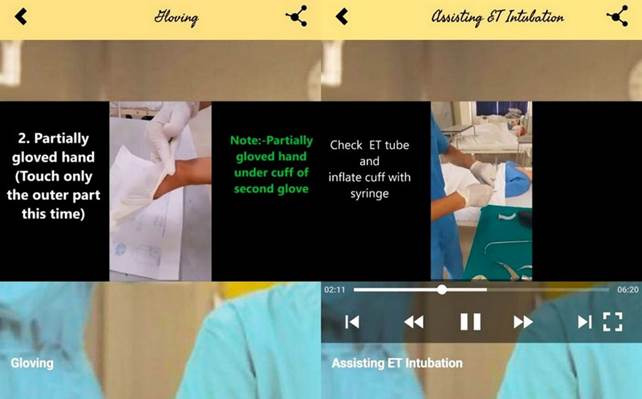
Screenshot of procedure videos (gloving and assisting in ET intubation)

Mobile Based Learning. Mobile Based Learning, which was developed as an intervention in this study, was created considering the learning requirements of nursing students. The application contains various sections, including videos, images, and electronic notes. (Figure 2-4) The following Mobile Based Learning on Operation Theatre techniques were chosen for this study: (i) Scrubbing: Introduction, purposes of scrubbing, and scrubbing procedures. The video is 3 minutes and 51 seconds in length; (ii) Gowning: Introduction, required gowning materials, gowning steps, and removal of personal protective equipment (PPE). The video lasts a total of 3 minutes and 17 seconds; (iii) Gloving: The introduction to gloving, the stages of gloving, and the removal of gloving. The video's duration is 2 minutes and 52 seconds; and (iv) Assisting ET Intubation: Introduction, purposes, procedures of Assisting ET Intubation, ET tube size, and how to secure ET tube. This video is 6 minutes and 19 seconds long. The researcher performed and recorded all procedure videos and then uploaded them to the application. The URL to the application was distributed to the nursing students' WhatsApp group. 

**Figure:**
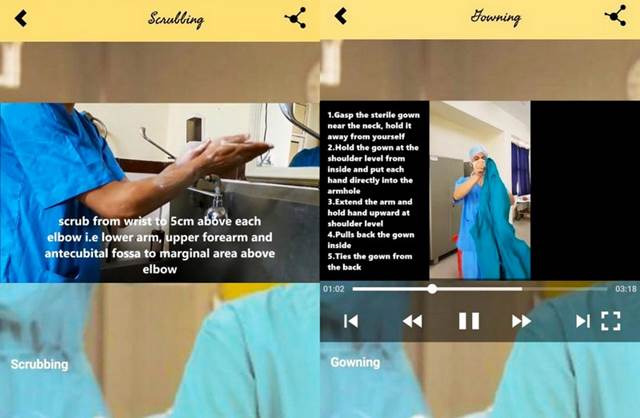

For data collection, instruments were created and divided into four sections. The first section consists of seven questions regarding selected variables of nursing students, including age, gender, interests, family income, residence, OT assignment, and prior E-Learning experience. The second section devised a structured questionnaire to assess nursing students' knowledge of procedures related to Operation Theatre. It comprised of 30 items (multiple choice questions) with each item having a single correct answer. Every correct answer was awarded a score of one and every wrong answer was awarded zero mark. The maximum and minimum possible score was 30 and zero respectively. The third section, Objective Structured Clinical Examination (OSCE) Questionnaire with observation checklist, was designed to evaluate the skills of nursing students in relation to operating room procedures. There were four OSCE stations of Scrubbing, Gowning, Gloving & Assisting in ET Intubation. There was a total of 46 items (Scrubbing: 15, Gowning: 11, Gloving: 10 and Assisting in ET Intubation: 10) on the observation checklist with a 3-point grading system. The maximum score for skills was 72 and minimum was zero. The Semantic Differential Scale Opinionnaire with ten items having alternative responses was designed to gauge nursing students' perspectives on the Satisfaction of Mobile-based Learning. The instrument’s content validity was evaluated by a panel of nine experts from the field of Medical Surgical Nursing, Anesthesiology, Nursing Educators and a Software Expert for its accuracy and applicability, and to solicit their feedback and recommendations. The CVI of tools were 0.8, 0.82 and 0.9 respectively for structured knowledge questionnaire, OSCE questionnaire and semantic differential scale. In addition, using Kuder-Richardson-20,^18^ the reliability of the structured knowledge questionnaire was 0.84. Inter-rater reliability for the OSCE questionnaire with observation checklist was 0.90 and Cronbach’s alpha ^19^ for satisfaction scale was 0.81.

Data Analysis. The data was analyzed for different parameters including descriptive statistics such as frequency, mean, standard deviation and inferential statistics including t-test, Mann Whitney U-test and Wilcoxon Signed Rank Tests. The normality of data was checked by Kolmogorov-Smirnov test. SPSS 20.00 software was used for data analysis. 

## Results

Since the data was normally distributed in terms of knowledge scores but not normally distributed for skills scores, thus both parametric and non-parametric tests were used for analysis. Participant Demographics. Most students in both the experimental and comparison groups were between the ages of 18-20 years, with 89.10% and 75%, respectively. Both the groups consisted primarily of females (85.90 and 73.40 percent, respectively). The percentage of nursing students who read for pleasure was lower than 50% in both the comparison (34.4%) and experimental (29.70%) groups. The median household income of the experimental group was 51.9 thousand rupees (708.56 USD), while that of the comparison group was 29.7 thousand (356.68 USD). In the comparison group, only 46.90% of the nursing students live at home, whereas 53.10% of the experimental group lives in a hostel. Sixty-nine percent of the nursing students both in comparison and experimental groups have not completed OT placement. 

Knowledge. The mean knowledge score of the nursing students was 13.03±3.285 in the experimental group, while in the comparison group it was 13.77±2.915, prior to the implementation of Mobile-based learning. In the post-test, the mean knowledge scores were found higher both in the experimental group (19.23±5.377; mean difference=-6.203) and in the comparison group (14.11±3.36; with a mean difference of 0.344). A paired "t" test was performed to compare the average knowledge scores within the two groups. The calculated "t" value of 8.337 in the experimental group was found to be statistically significant (t (63) = 2.000; p<0.001) at the 0.05 level of significance; in contrast, the calculated "t" value of 0.59 in the comparison group was not statistically significant (t (63) = 2.000; p=0.551). For comparing the mean difference between the experimental and comparison group, unpaired t-test was applied, and the calculated t value of 6.46 (t (126) =1.98; p<0.001) was found to be statistically significant in the post-test. (Table 1)

**Table 1:** Comparison of Knowledge of Nursing students

Variable	Experimental Group (n=64)	Comparison group (n=64)	t-value	df	p-value
Pre-test Knowledge	13.03±3.28	13.77±2.91	1.338	126	0.191
Post-test Knowledge	19.23±5.37	14.11±3.36	6.46	126	<0.001
t-value	8.337	0.59			
DF	63	63			
p-value	<0.001	0.551			

Skills. To compare the skills of nursing students between the groups, Mann-Whitney U test was applied and the results revealed that the mean ranks of skills of students in the experimental group were significantly higher in all areas i.e., Scrubbing, Gowning, Gloving and for Assisting in ET Intubation. (Table 2)

**Table 2:** Comparison of nursing students in terms of level of skills after administration of mobile based learning

Skills	Group	Mean rank	Mann-Whitney U	Z-value	p-value
Scrubbing	Experimental (n=64)	94.57	123.500	-9.192	<0.001
	Comparison (n=64)	34.43			
Gowning	Experimental (n=64)	91.92	293.000	-8.400	<0.001
	Comparison (n=64)	37.08			
Gloving	Experimental (n=64)	78.59	1146.000	-4.325	<0.001
	Comparison (n=64)	50.41			
Assisting ET Intubation	Experimental (n=64)	73.91	1445.500	-2.882	0.004
	Comparison (n=64)	55.09			

The Wilcoxon Signed Rank test was used to determine the rank, rank sum, and Z value of nursing students in the experimental group's pre- and post-test scores on competencies relevant to operating room procedures. The Post-test scores for the skills of Scrubbing (20.52), Gowning (16.70), Gloving (13.77), and assisting in ET Intubation (10.58) were all significantly higher after the administration of mobile based learning. The mean ranks for Scrubbing (33.95), Gowning (33.77), Gloving (33.57), and Assisting ET Intubation (32.74) were all found to be significant (p<0.001). (Table 3)

The satisfaction of nursing students regarding mobile based learning was mostly high (93.7%), and the rest was moderate (6.3%). 

**Table 3:** Comparison of nursing students in terms of level of skills before and after administration of Mobile Based Learning

Skills	Group	Mean ±SD	Mean rank	Z-value	p-value
Scrubbing	Pre-test	11.69±4.18	33.95	-6.725	<0.001
	Post-test	20.52±3.30			
Gowning	Pre-test	11.84±3.02	33.77	-6.421	<0.001
	Post-test	16.70±2.64			
Gloving	Pre-test	9.66±3.86	33.57	-4.726	<0.001
	Post-test	13.77±3.45			
Assisting ET Intubation	Pre-test	6.30±4.77	32.74	-4.841	<0.001
	Post-test	10.58±4.51			

## Discussion

The mean knowledge and skills scores of nursing students were significantly higher after the administration of Mobile Based Learning. Moreover, learning on mobile application gave the students an opportunity to repeat the steps or skills whenever and wherever they want to. When these results were analyzed with previous researches, consistency in results were shown in the studies ^9^,^20^ which demonstrated that the mean knowledge and skills scores were significantly higher after the intervention than before it. In another study conducted on the blood pressure measurement skills of nursing students, the mobile application was found to be effective in improving the skills.^21^ Furthermore, it was noted in multiple studies that the utilization of mobile devices and videos resulted in an enhancement of nursing students' knowledge and skills.^22^-^24^

In the present study, there were significant differences between pre-test and post-test knowledge and skills scores among nursing students. This was consistent with the results of a study where it was discovered that the knowledge score and skills score of students increased after the administration of intervention.^21^ In contrast, it was found in another study that nursing students' knowledge increased, but it was not statistically significant (p=0.379).^12^ Although, the difference is knowledge scores was not significant, but there was a positive trend focusing on the development of theoretical understanding as well. This could be attributed to certain factors like baseline knowledge level, and the nature of intervention emphasizing on skill acquisition. A substantial difference was observed in the scores of pre-test knowledge and post-test knowledge as well as in the scores of scrubbing skills, gowning skills, gloving skills and assisting in ET intubation in the experimental group before and after the administration of Mobile Based Learning. In accordance with another study’s findings, there was a highly statistically significant difference between pre and post knowledge scores (t = 8.845, p<0.001) and skills scores (t = 7.471, p<0.001) before and after the administration of the Intervention.^15^

The nursing students in the present study were highly satisfied with the mobile based learning intervention owing to the fact that they were able to access the procedures on the application at their own time and could watch the videos any number of times without any restriction. Other studies carried out using mobile technology have found similar results where the students found it more comfortable learning the skill-based procedures through technology.^25^-^27^

One of the limitations of this study was that the researchers did not monitor the number of times, students watched the videos, which might have an effect on their scores. Another one is, since the mobile application is exclusively compatible with android phones, the study included only those students who possessed a phone with this system. In future, similar application with enhanced compatibility with all devices may be developed. Other significant nursing procedures may also be taught through these kinds of applications making a comfortable learning environment for the students as well as for new nurses. The findings have implications in every facet of nursing be it education, practice, administration, or research. A certain percentage of every nursing course shall be delivered through mobile based learning approach. Nurses or nursing students can make informed decisions by utilizing knowledge obtained from either the textbooks or the mobile app. And these days, utilizing the applications on mobiles is more accessible and gives a flexible platform for learning, and is thus preferred by most of the students.

Conclusion. The results of present study conclude that Mobile Based Learning is an effective method for improving both knowledge and skills of nursing students. Therefore, the use of mobile based learning is strongly recommended to assist the learners in improving their abilities besides knowledge about significant procedures.
